# Physical Education Fallacies

**Published:** 1882-02

**Authors:** 


### PHYSICAL EDUCATION FALLACIES.

1. The Leading-String Fallacy.—From the moment a child is born, he is treated on the principle that all his instincts are essentially wrong, that Nature must be thwarted and counteracted in every possible way. He is strapped up in a contrivance that he would be glad to exchange for a strait-jacket, kept for hours in a position that prevents him from moving any limb of his body. His first attempts at locomotion are checked; he is put in leading-strings, he is carefully guarded from the out-door world, from the air that would invigorate his lungs, from the sports that would develop his muscles. Hence, the peevishness, awkwardness, and sickliness of our young aristocrats. Poor people have no time to imitate the absurdities of their wealthy neighbors, and their children profit by what the model nurse would undoubtedly call neglect. Indian babies are still better off. They are fed on bull-beef, and kicked around like young dogs; but they are not swaddled, they are not cradled, and not dosed with paregoric; they crawl around naked, and soon learn to keep out of the way; they are happy, they never cry. If we would treat our youngsters in the same way, only substituting kisses and bread for kicks and beef, they would be as happy as kids in a clover-field, and moreover they would afterward be hardier and stronger. Every week the newspapers tell us about ladies tumbling down-stairs and breaking both arms; boys falling from a fence and fracturing their collar-bones. From what height would a young Comanche have to fall to break such bones—not to mention South-Sea Island children and young monkeys? The bones of an infant are plastic: letting it tumble and roll about would harden the bone tissue; guarding it like a piece of brittle crockery makes its limbs as fragile as glass. Christian mothers reproach themselves with neglecting their duty to their children if they do not constantly interfere with their movements, but they forget that in points of physical education Nature herself is such an excellent teacher that the apparent neglect is really a transfer of the pupil to a more efficient school.

2. The Nostrum Fallacy.—When a child complains of headache, lassitude, or want of appetite, the nurse concludes that he must "take something." If the complexion of a young lady grows every day paler and pastier, her mother will insist that she must "get something" to purify her blood. If the baby squeals day and night, a doctor is sent for, and is expected to "prescribe something." What that something should be, the parents would be unable to define, but they have a vague idea that it should come from the drug-store, and that it cannot be good for much unless it is bitter or nauseous. Traced to its principles their theory would be about this: "Sickness and depravity are the normal condition of our nature; salvation can come only through abnormal agencies; and a remedy, in order to be effective, should be as anti-natural as possible." Perfectly logical from a Scriptural point of view. But Nature persists in following her own laws. Her physiological laws she announces by means of the instincts which man shares with the humblest of his fellow-creatures, and health is her free gift to all who trust themselves to the guidance of those instincts. Health is not lost by accident, nor can it be repurchased at the drug-store. It is lost by physiological sins, and can be regained only by sinning no more. Disease is Nature's protest against a gross violation of her laws. Suppressing the symptoms of a disease with drugs means to silence that protest instead of removing the cause. We might as well try to extinguish a fire by silencing the fire-bells; the alarm will soon be sounded from another quarter, though the first bells may not ring again till the belfry breaks down in a general conflagration. For the laws of health, though liberal enough to be apparently plastic, are in reality as inexorable as time and gravitation. We can only bully Nature, we can not defy her resentment by a fresh provocation. Drugs may change the form of the disease—i. e., modify the terms of the protest —but the law cannot be baffled by complicating the offense; before the drugged patient can recover, he has to expiate a double sin—the medicine and the original cause of the disease. But shall parents look on and let a sick child ask in vain for help? By no means. Something is certainly wrong, and has to be righted. The diasease itself is a cry for help. But not for drugs. Instead of "*taking* something," something ought to be *done*, and oftener something habitually done ought to be *omitted*. If the baby's stomach has been tormented with ten nursings a day, omit six of them; omit tea and coffee from the young lady's *menu;* stop the dyspeptic's meat-rations, and the youngster's grammar-lessons after dinner. But open the bedroom-windows, open the door and let your children take a romp in the garden, or on the street, even on a snow-covered street. Let them spend their Sundays with an uncle who has a good orchard; or, send for a barrel of apples. Send for a carpenter, and let him turn the nursery or the wood-shed into a gymnasium. In case you have nothing but your bedroom and kitchen, there will still be room for a grapple-swing; the Boston Hygienic Institute has patented a kind that can be fastened without visible damage to the ceiling. If the baby won't stop crying, something ought to be done about it. Yes, and as soon as possible: remove the strait-jacket apparatus, swaddling-clothes, petticoat, and all, spread a couple of rugs in a comfortable corner, and give the poor little martyr a chance to move his cramped limbs; let him roll, tumble, and kick to his heart's content, and complete his happiness by throwing the paregoric-bottle out of the window.

3. The Stimulant Fallacy.—Eight hours of healthy sleep are sufficient to restore the energy expended in an ordinary day's work. Extraordinary efforts, emotional excitement, sensual excesses, or malnutrition (either by insufficient food or dyspeptic habits), induce a general lassitude—a warning that the organism is being overtasked. Repose and a healthier or more liberal diet will soon restore the functional vigor of the system. But during such periods of their diminished activity the vital powers can be rallied by drastric drugs or tonic beverages—in other words, by poisons. The prostrate vitality rises against a deadly foe, as a weary sleeper would start at the touch of a serpent; and, as danger will momentarily overcome the feeling of fatigue, the organism labors with restless energy till the poison is expelled. This feverish reaction, dram-drinkers (patent dram-drinkers especially) mistake for a sign of returning vigor, persistently ignoring the circumstance that the excitement is every time followed by a prostration worse than that preceding it. Feeling the approach of a relapse the stimulator then resorts to his old remedy, thus inducing another sham revival, followed by an increased prostration, and so on; but before long the dose of the stimulant, too, has to be increased, the stimulator becomes a slave to his poison, and passes his life in a round of morbid excitements and morbid exhaustions—the former at last nothing but a feeble flickering-up of the vital flame, the latter soon aggravated by sick headaches, "vapors," and hypochondria.

The stimulant habit in all its forms—"exhilarating beverages," "tonic medicines," "prophylactic bitters," etc.—is a dire delusion. A healthy man needs no artificial excitants; the vital principle in its normal vigor is an all-sufficient stimulus; the inspiration bought at the rum-shop is but a poor substitute for the spontaneous exaltatations of a healthy mind in a healthy body. Playing with poisons is a losing game; the sweetness of the excitement is not worth the bitter reaction. In sickness stimulants can not further the actual recovery by a single hour. There is a strong progressive tendency in our physical constitution; Nature needs no prompter: as soon as the remedial process is finished, the normal functions of the organism will resume their work as spontaneously as the current of a stream resumes its course after the removal of an obstruction. A "prophylactic" brandy is Old Scratch in the *role* of an exorcist.

Fevers can be prevented by other means; and at any rate the possible danger of a climatic disease is preferable to the sure evils of the poison-drug. But how can noxious stimulants be distinguished from wholesome drinks? Tonic medicines, stimulating beverages, and poisons, are synonymous terms. Every known poison can become a lusted-after stimulant by forcing it repeatedly upon the (at first) reluctant stomach. It is true that the hankering of an old *habitue* after his tipple resembles the craving of a hungry man for food, but that constitutes no reproach against Nature, for the taste of the first drink betrayed the poison. To the palate of a child narcotic stimulants are bitter, alcohol is burning-acrid, tobacco nauseous, mineral poisons either bitter or insipid. By a liberal admixture of sugar and milk the repulsiveness of various narcotic decoctions can be diminished, but in no disguise could they be possibly mistaken for nourishing substances if the natural depravity dogma had not weakened our confidence in the testimony of our instincts.—*Popular Science Monthly.*

